# Fighting with the Lernean Hydra: Greek Humanitarian Crisis Enters Worst-Case Scenario. Comment on: Joseph, L.; Ismail, S.A.; Gunst, M.; Jarman, K.; Prior, D.; Harris, M.; Abbara, A. A Qualitative Research Study Which Explores Humanitarian Stakeholders’ Views on Healthcare Access for Refugees in Greece. *Int. J. Environ. Res. Public Health* 2020, *17*, 6972

**DOI:** 10.3390/ijerph17218253

**Published:** 2020-11-09

**Authors:** Ourania S. Kotsiou, Panagiotis Kotsios, Konstantinos I. Gourgoulianis, Vaios Kotsios

**Affiliations:** 1 Department of Respiratory Medicine, Faculty of Medicine, University of Thessaly, BIOPOLIS, 41110 Larissa, Greece; kgourg@med.uth.gr; 2 School of Social Sciences, Hellenic Open University, 26335 Patras, Greece; panagiotiskotsios@gmail.com; 3 Metsovion Interdisciplinary Research Center, National Technical University of Athens, 44200 Athens, Greece; vaioskotsios@gmail.com

Liz Joseph and collaborators shed light upon the real challenges of securing health during the Greek humanitarian crisis from the point of view of the key stakeholders in healthcare access, reflecting the need to reform a range of different contexts and types of humanitarian response [1]. The humanitarian crisis that has vexed the world community hitherto still resembles the Lernean Hydra. The Greek case provides a remarkable paradigm of instability within an unabated political fog due to the refugee crisis, tense Greek–Turkish relations, deepening economic and human rights problems, and the coronavirus disease 2019 pandemic crisis.

After a prolonged and deep recession, the Greek economy started to grow again from late 2016; but even labor market recovery has been a slow process. Greece’s gross domestic product fell by 22% in 11 years, to 184 billion euros in 2018 [2]. Total health expenditure dropped from 22.49 billion euros in 2009 to 14.49 billion euros in 2017 [2]. After peaking in 2008, health spending per capita declined by almost a third over the following years, reaching 1347 euros in 2017, 45% less than the European average [2].

The economic consequences of the lockdown are the epicenter of the crisis in most countries, especially in nations which were already hard-hit by the economic recession preceding the pandemic crisis. The pandemic recession is leaving clear traces on the labor market and on the national budget in Greece. According to the International Monetary Fund, the Greek economy is expected to shrink by 10% of gross domestic product (GDP) in the current year because of the coronavirus pandemic and its devastating effects on domestic and global economies. With the Greece 2020 tourism season looming, Greece faces several serious challenges that could end up stifling industrial growth.

Meanwhile, Greece has been at the epicenter of the refugee crisis. In 2018, 61,460 refugees and 76,099 asylum seekers arrived in Greece, almost double the number recorded the year before [3]. The estimated cost of the refugee crisis to public expenditure for 2016 was about 600 million euros [3]. Public health facilities in the reception centers on Lesvos and Chios have become more overcrowded since the end of 2017 (Figure 1). The short average length of hospital stay may mask the real extent of overcrowding, which is even more evident at the clinic level.

A robust paradigm of the nation’s healthcare dysfunction can be seen in the unique Neurosurgery Department (Lesvos), where bed coverage exceeded 100% in 2020, and was more than half of that in 2016–2018. Greece lacks mechanisms to allow planning and optimal allocation of physical and human resources [4,5]. The struggle for global assistance should be the core principle of humanitarian crisis management [3].

Data were obtained from the Ministry of Health’s database. There is no copyright issue.

## Figures and Tables

**Figure 1:**
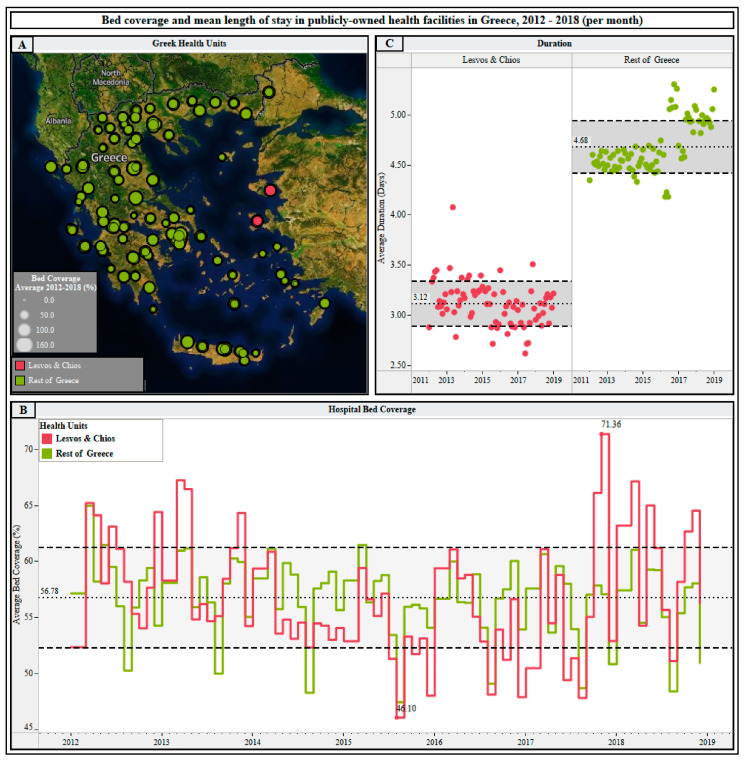
A visual comparison of bed coverage and length of hospital stay between publicly-owned health facilities in main reception centers on the Aegean islands of Lesvos and Chios and the rest of Greece, 2012–2018. (**A**): The red circles represent bed coverage in the main reception centers on Lesvos and Chios per publicly-owned hospital, 2012–2018; the green circles represent bed coverage in publicly-owned health facilities on the mainland. The area of the circle depicts the mean percentage of bed coverage per publicly-owned hospital in Greece, 2012–2018; (**B**): Variance of the mean bed occupancy rate in publicly-owned health facilities in the main reception centers on Lesvos and Chios (red line) and publicly-owned health facilities on the mainland (green line) per month for the period 2012–2018. The mean overall bed occupancy rate for the period 2012–2018 was 56.78 ± 4.48% in Greece. The mean bed occupancy rate in Lesvos and Chios has been greater than the overall Greek mean bed occupancy rate since the end of 2017; (**C**): Red points represent the mean length of hospital stay (days) in publicly-owned health facilities in main reception centers on Lesvos and Chios per month for the period 2012–2018 (mean length of stay over the entire period: 3.12 ± 0.23 days). Green points represent the mean length of hospital stay (days) in publicly-owned health facilities in the rest of Greece per month for the period 2012–2018 (mean length of stay over the entire period: 4.68 ± 0.26 days).
